# *Schisandra* extract ameliorates arthritis pathogenesis by suppressing the NF‐κB and MAPK signalling pathways

**DOI:** 10.1111/jcmm.17814

**Published:** 2023-06-20

**Authors:** Seong Jae Han, Hyemi Lee, Jiho Nam, Cheol‐Ho Pan, Jimin Jeon, Siyoung Yang

**Affiliations:** ^1^ Department of Biomedical Sciences Ajou University Graduate School of Medicine Suwon Republic of Korea; ^2^ Department of Biological Sciences Sungkyunkwan University Suwon Republic of Korea; ^3^ Natural Product Informatics Research Center Korea Institute of Science and Technology Gangneung Republic of Korea

**Keywords:** arthritis, cartilage destruction, catabolic factor, *Schisandra* extract

## Abstract

*Schisandra chinensis* is a medicinal plant used to treat various diseases. Extracts from the leaves or fruits of *S. chinensis* and their components are used in osteoarthritis (OA). The OA inhibitory effect of schisandrol A, one of its components, has been previously confirmed. We aimed to confirm the OA inhibitory effect of *Schisandra* (including components like schisandrol A) to identify why the inhibitory effect of the *Schisandra* extract is better. First, we investigated the effects of the *Schisandra* extract on OA as a potential therapeutic. Experimental OA was induced in a mouse model via destabilized medial meniscus surgery. The animals were orally administered the *Schisandra* extract; the inhibition of cartilage destruction was confirmed using histological analysis. In vitro analysis showed that the *Schisandra* extract attenuated osteoarthritic cartilage destruction by regulating IL‐1β‐induced MMP3 and COX‐2 levels. The *Schisandra* extract inhibited IL‐1β‐induced degradation of IκB (NF‐κB pathway) and IL‐1β‐induced phosphorylation of p38 and JNK (mitogen‐activated protein kinase (MAPK) pathway). RNA‐sequencing analysis showed that the *Schisandra* extract decreased the expression of IL‐1β‐induced MAPK and NF‐κB signalling pathway‐related genes more than schisandrol A alone. Therefore, *Schisandra* extract may be more effective than schisandrol A in preventing OA progression by regulating MAPK and NF‐κB signalling.

## 1 INTRODUCTION

Osteoarthritis (OA) is a joint disorder that may affect multiple joints. The anatomical perturbations observed in OA include cartilage degradation, joint stiffness, pain and swelling.^1^ The pathological mechanism of cartilage destruction in OA involves the upregulation of catabolic factors, such as matrix metalloproteinases (MMPs), enzymes that promote cartilage degradation, and COX‐2, which are mainly associated with inflammation.^2^, ^3^ The levels of these catabolic factors are increased by pro‐inflammatory cytokines, such as interleukin (IL)‐1β, which plays a vital role in cartilage degradation and is involved in OA development.^4^, ^5^ These catabolic factors are expressed via NF‐κB and mitogen‐activated protein kinase (MAPK) signalling pathways.^6^ NF‐κB is involved in biological processes, including inflammation and cell proliferation and differentiation.^7^, ^8^ Previous studies have reported that the NF‐κB signalling pathway is associated with the development of OA via the downstream activation of catabolic factors in articular chondrocytes, such as the increase in expressions of MMPs and COX‐2.^9^, ^10^ In addition, MAPK subtypes (extracellular signal‐regulated kinases1/2 [ERK1/2], p38, c‐Jun N‐terminal kinase [JNK]) are known to produce MMPs and COX‐2 in chondrocytes and are involved in the progression of OA.^11^, ^12^ Thus, NF‐κB and MAPK signalling pathways are activated by IL‐1β, which increases the expression of catabolic factors such as MMP3 and COX‐2, leading to the onset of OA.^13^

Currently, natural plant‐based treatments are being developed as alternatives for several diseases because they have fewer side effects than previously known drugs.^14^, ^15^ Previous studies have shown that extracts from some plants inhibit the development of OA.^16^, ^17^ *Schisandra chinensis* (Turcz.) Baill is cultivated in northeastern China, Korea, and Japan. As a traditional Chinese herb, it has been used as an antitussive and for the treatment of insomnia and hepatitis.^18^, ^19^, ^20^ Recent studies have shown that the extracts of *Schisandra* possess biological activities, including anticancer, antioxidant, neuroprotective, hepatoprotective and anti‐inflammatory activities.^21^ In addition, studies have reported that the *Schisandra* leaf extract relieves pain and inflammation in a rat model of OA.^22^, ^23^

*Schisandra* is composed of several single compounds, one of which is schisandrol A, which is known to exert various pharmacological effects, including antioxidant, antiapoptotic and antiallergic effects.^24^ Previous studies have shown that schisandrol A suppresses pro‐inflammatory mediators (e.g. COX‐2), which inhibit NF‐κB activation.^24^ In our previous study, we showed that schisandrol A inhibits OA progression by inhibiting NF‐κB signalling.^25^

Here, we aimed to investigate whether the use of the *Schisandra* extract containing various biologically active compounds affects OA progression more than that of a single substance, such as schisandrol A. To achieve this, we set up in vitro OA conditions using chondrocytes and compared the extent of changes in the expression of signalling genes involved in OA after treatment with schisandrol A (single compound) and *Schisandra* extracts containing various biologically active substances.

## 2 MATERIALS AND METHODS

### 2.1 Mouse model

The in vivo animal experiments were approved by the Animal Care and Use Committee of Ajou University, and the procedures adhered to the 8th edition of the Guide for the Care and Use of Laboratory Animals issued by the National Institutes of Health (protocol code 2016–0041). C57BL/6 mice and 5‐day‐old Institute of Cancer Research (ICR) mice were purchased from DBL Co. Ltd. C57BL/6J male mice weighing 18–20 g (10 weeks old) were housed at 23°C and exposed to a 12/12‐h light–dark cycle; water and food were supplied regularly. Five‐day‐old ICR mice were used for articular primary chondrocyte cultures, and C57BL/6 mice were used for setting up the destabilized medial meniscus (DMM) mouse model.

### 2.2 Primary culture of articular chondrocytes and viability analysis

Articular chondrocytes were obtained from the femoral and tibial plateaus of 5‐day‐old postnatal ICR mice. Cartilage tissue was digested using 0.2% collagenase type II enzyme. The chondrocytes were seeded in 96‐well plates (9 × 10^3^ cells/well) and incubated for 48 h before treatment with *Schisandra* and schisandrol A. The articular chondrocyte primary cultures were incubated in Dulbecco's Modified Eagle's Medium (DMEM) supplemented with 10% fetal bovine serum (Capricon) and 1% penicillin–streptomycin (Capricon, Ebsdorfergrund, Germany). Different concentrations of *Schisandra* (50, 100, 250, 500 and 1000 μg/mL) were used for 24 h. Cell viability was analysed using the culture medium and a lactate dehydrogenase (LDH) colorimetric assay kit (BioVision, Inc.). Triton X‐100‐treated (viability, 0%) and untreated samples (viability, 100%) were used for normalization. Viability was determined using the following formula: 100 − (sample LDH − negative control)/(maximum LDH − negative control) × 100. Each signal was measured at 495 nm using a Synergy H1 microplate reader (Biotek).

### 2.3 Reagents and treatment

Schisandrol A was purchased from Sigma–Aldrich. IL‐1β was purchased from GenScript. Schisandrol A was dissolved in dimethyl sulfoxide for in vitro analysis, and the IL‐1β recombinant protein was dissolved in sterile water. For oral administration to mice, various concentrations of schisandrol A (50, 100 and 500 mg/kg) were dissolved in phosphate‐buffered saline (PBS). To induce OA in vitro, mouse articular chondrocytes were treated with IL‐1β (1 ng/mL) or co‐treated with IL‐1β and *Schisandra* (50, 100 and 250 μg/mL) or schisandrol A (1000 μM, positive control) for 24 h prior to harvesting.

### 2.4 Quantitative reverse transcription‐polymerase chain reaction (qRT‐PCR)

Total RNA was obtained from mouse articular chondrocytes using TRIzol reagent (Molecular Research Center Inc.). The sequences of primers used (for MMP, GAPDH and COX‐2) are shown in Table S1. The level of target gene amplification was determined using qRT‐PCR with SYBR® Green fluorescence and Premix Ex Taq (TaKaRa Bio). The transcription level of each target gene was normalized to that of *GAPDH* and expressed as the fold change relative to the control group.

### 2.5 Protein isolation and western blotting

Total protein was extracted from primary cultured chondrocytes using radioimmunoprecipitation assay lysis buffer containing 150 mM NaCl, 1% NP‐40, 50 mM Tris/HCl (pH 8.0), 0.2% sodium dodecyl sulphate (SDS) and 5 mM NaF with a protease and phosphatase inhibitor mixture (Roche, Madison, WI, USA). Whole proteins were separated via SDS‐polyacrylamide gel electrophoresis, and western blotting was performed. The following antibodies were used: goat anti‐COX‐2 (sc‐1745; Santa Cruz Biotechnology) and mouse anti‐ERK1/2 (610408; Becton Dickinson). mouse anti‐ERK1/2 (610,408; Becton Dickinson), mouse anti‐IκB (9242; Cell Signaling Technology [CST]), mouse anti‐p65 (8242; CST), mouse anti‐phospho‐p65 (3033; CST), mouse anti‐p38 (#9212; CST), mouse anti‐pp38 (#9215S; CST), mouse anti‐JNK (#9252S; CST), mouse anti‐pJNK (#9251S; CST), mouse anti‐pERK (#9101S; CST). Each signal was visualized using a SuperSignal West Dura kit (Thermo Scientific) according to the manufacturer's instructions. Densitometric analysis (AlphaEase FC 4.0; Alpha Innotech) was used to calculate relevant band intensities. ERK was used as the loading control.

### 2.6 Prostaglandin E2 (PGE_2_ ) and collagenase assays

PGE_2_ expression was determined using a PGE_2_ immunoassay kit (R & D Systems). The levels of PGE_2_ were measured in the conditioned medium of articular chondrocytes. Collagenase activity in the conditioned medium of the articular chondrocyte culture was assessed using the EnzCheck Gelatinase/Collagenase Assay Kit (Molecular Probes) and a VICTOR X3 microplate reader (PerkinElmer) at excitation/emission wavelengths of 490/530 nm, following the manufacturer's protocol.

### 2.7 Mouse model of experimental OA and oral administration of the extract

To create an OA model via medial meniscus tear, we performed DMM surgery to cut the meniscus of the mouse knee joint in 10‐week‐old male C57BL/6J mice. The mouse knee joint was processed for histological analysis 10 weeks after surgery. In the DMM‐induced OA model, *Schisandra* (100, 200 or 500 mg/kg) was administered orally every other day for 6 weeks, and the mice were euthanized at the end of the 6‐week regimen. Four treatment groups (DMM + PBS, DMM + 5 mg/kg extract, DMM + 10 mg/kg extract and DMM + 50 mg/kg extract) were used, with five animals in each treatment group.

### 2.8 Evaluation of cartilage destruction

Cartilage destruction was evaluated via safranin O staining and scored using the Osteoarthritis Research Society International (OARSI) grading system. Mouse knee joints were fixed using 4% paraformaldehyde, dehydrated using 0.5 M EDTA (pH 8.0) for 2 weeks and embedded in paraffin. The paraffin blocks were cut into 5 μm sections and fixed on glass slides. The sections were hydrated using a graded ethanol series, and xylene was used to remove paraffin. Immunohistochemistry was performed on mouse knee joint sections using anti‐COX‐2 (66351‐I‐Ig, Protein Tech).

### 2.9 Culture of cartilage explants and Alcian blue staining

*Schisandra* extract (50, 100 and 250 μg/mL) or schisandrol A (400, 800 and 1000 μM) was administered to cartilage explants isolated from the knee joints of 5‐day‐old ICR mice (DBL) in combination with IL‐1β for 48 h in DMEM. The cartilage explants were fixed using 4% paraformaldehyde, dehydrated using different concentrations of ethanol and embedded in paraffin blocks. The paraffin blocks were then cut into pieces of 5 μm thickness. The accumulation of sulphated proteoglycans was determined using Alcian blue staining.^14^

### 2.10 Statistical analysis

The data are presented as mean ± standard error of the mean. Two researchers independently prepared all the histological samples. Each experiment was performed at least five times. One‐way analysis of variance (anova) with Bonferroni's post hoc test was used for data analysis. The Prism 7 software (https://www.graphpad.com/scientific‐software/prism/) was used to perform statistical analyses, and significance was defined at *p* ≤ 0.05.

## 3 RESULTS

### 3.1 *Schisandra* extract was not cytotoxic for chondrocytes

**FIGURE 1:**
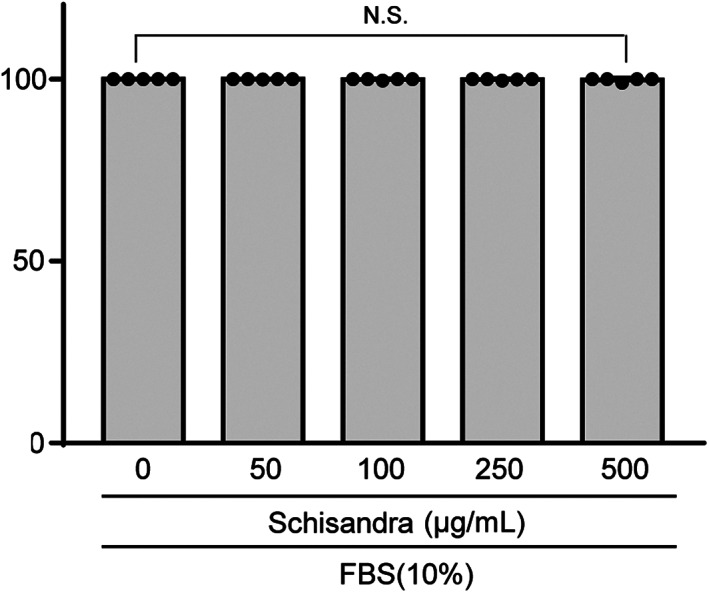
Toxicity of *Schisandra* to chondrocytes. Cell viability was measured at various concentrations for 24 h and analysed using a lactate dehydrogenase (LDH) assay. Data were analysed using one‐way analysis of variance with Bonferroni's test, and the plotted values are indicated as mean ± standard error of the mean.

First, we examined the cytotoxic effects of the *Schisandra* extract on chondrocytes. Chondrocytes were treated with 0, 50, 100, 250 and 500 μg/mL of the *Schisandra* extract for 24 h. As shown in Figure 1, the viability of the chondrocytes was not affected by any of the concentrations of the *Schisandra* extract when compared with that of the control. Based on these observations, all subsequent experiments were performed using 50, 100 and 250 μg/mL of the extract for 24 h.

### 3.2 *Schisandra* extract suppressed the IL‐1β‐induced expression of catabolic factors in articular chondrocytes

**FIGURE 2:**
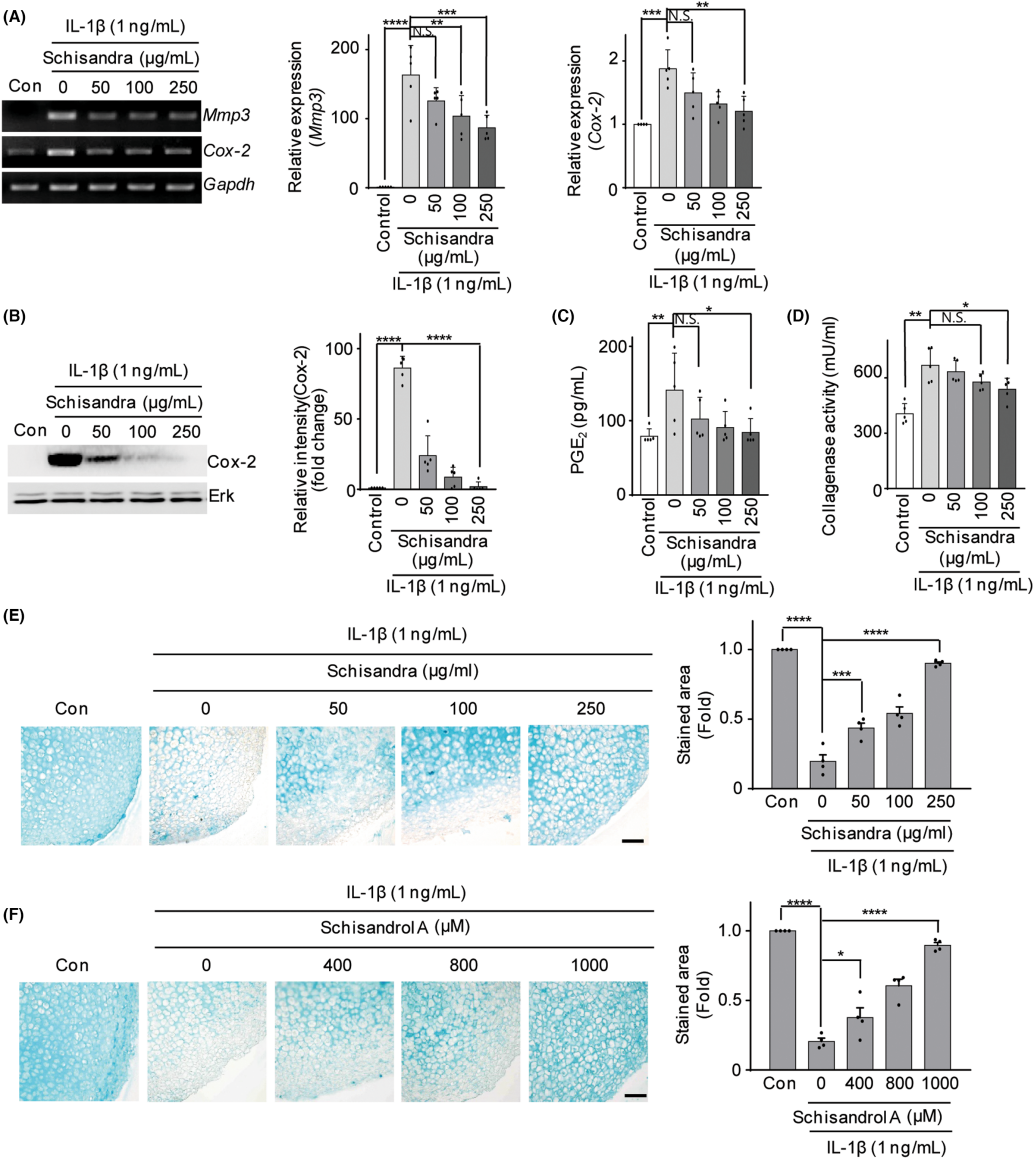
*Schisandra* inhibited the expression of MMPs and COX‐2, and reduced IL‐1β‐induced production of PGE_2_ and collagenase in mouse articular chondrocytes. Chondrocytes stimulated with IL‐1β (1 ng/mL) were treated with different concentrations of *Schisandra* (0, 50, 100 or 250 μg/mL). The mRNA levels of MMP3 and COX‐2 were measured using RT‐PCR and qRT‐PCR (A). COX‐2 protein level was determined using western blotting and densitometry (B). PGE_2_ (C) and collagenase (D) assays were performed with chondrocytes treated with various concentrations (0, 50, 100 or 250 μg/mL) of *Schisandra* after stimulation with IL‐1β (1 ng/mL). Glyceraldehyde 3‐phosphate dehydrogenase (GAPDH) and extracellular signal‐regulated kinase (ERK) were used as loading controls. Ex vivo cultured cartilage was co‐treated with IL‐1β and *Schisandra* extract (0, 50, 100 and 250 mg/mL) or schisandrol A (0, 400, 800 and 1000 μM) for 48 h and the cartilage area was analysed using Alcian blue staining (left); the stained area was quantified (right) (E, F). Data were analysed using one‐way analysis of variance with Bonferroni's test, and the plotted values are indicated as mean ± standard error of the mean; *p < 0.05, **p < 0.01, ***p < 0.001 and ****p < 0.0001 compared to the control group. Scale bar = 100 μm.

We have already shown that the expression of catabolic factors such as MMP3 and COX‐2 induces cartilage destruction.^9^, ^10^ Therefore, we examined whether the *Schisandra* extract suppressed the expression of these catabolic factors. When chondrocytes were co‐treated with the *Schisandra* extract and IL‐1β for 24 h, the mRNA expression levels of MMP3 and COX‐2 first increased under the influence of IL‐1β and then gradually decreased as the concentration of *Schisandra* increased, as determined using RT‐PCR (Figure 2A, left panel) and qRT‐PCR (Figure 2A, right panel). At the protein level, a similar decrease in the expression levels of COX‐2 was determined using western blotting (Figure 2B; left panel) and densitometric analysis (Figure 2B; right panel). In addition, we performed experiments to examine whether the *Schisandra* extract inhibited MMP activity. MMP3 possesses collagenase activity and reduces the contents of aggrecan, type II collagen, and other components of the extracellular matrix (ECM).^9^, ^10^ The *Schisandra* extract considerably reduced the increase in collagenase activity induced by IL‐1β in chondrocytes (Figure 2C). Furthermore, the *Schisandra* extract inhibited IL‐1β‐induced PGE_2_ production by chondrocytes (Figure 2D). In the ex vivo experimental conditions of OA created using IL‐1β, the degree of ECM degradation of the explant cartilage treated with IL‐1β was reduced after treatment with the *Schisandra* extract and schisandrol A, as observed using Alcian blue staining (Figure 2E,F). These results indicated that the *Schisandra* extract has the potential to block osteoarthritic progression by reducing MMP3 and COX‐2 levels.

### 3.3 Oral administration of the *Schisandra* extract suppressed cartilage destruction in the DMM‐induced arthritis model

**FIGURE 3:**
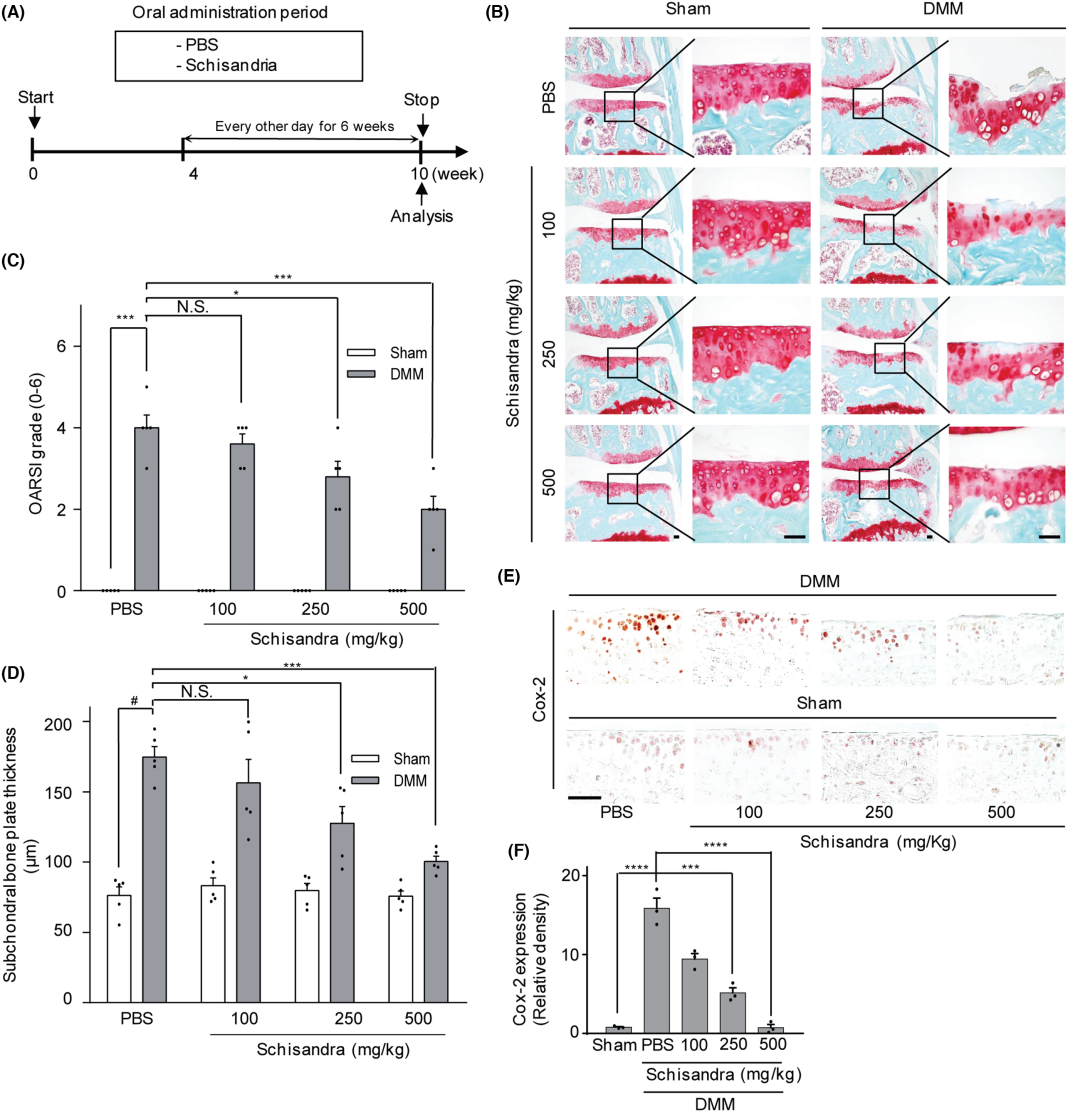
Oral administration of *Schisandra* suppressed cartilage destruction in osteoarthritis (OA). (A) Experimental plan for analysis of DMM‐induced arthritis. Mice were administered phosphate‐buffered saline (PBS) or *Schisandra* (100, 250 or 500 mg/kg) every other day for 4–10 weeks after DMM surgery until analysis. (B, C, D) Cartilage destruction was determined using Safranin‐O staining (B) Osteoarthritis Research Society International (OARSI) score (C), and subchondral bone plate thickness (D) at 10 weeks after DMM surgery. (E, F) COX‐2 expression was detected by Immunohistochemistry (E) and densitometry analysis (F). (C, D, F) Data were analyzed using one‐way analysis of variance with Bonferroni's test, and the plotted values are indicated as means ± standard error of the mean; *p < 0.05, ***p < 0.001, and ****p < 0.0001. Scale bar = 100 μm.

To examine whether oral administration of *Schisandra* suppressed arthritic cartilage degradation in vivo, we assessed the effect of the *Schisandra* extract in a mouse model of DMM‐induced OA. The *Schisandra* extract in PBS (or the PBS‐alone control) was orally administered to the mice thrice a week for 10 weeks after the DMM surgery (Figure 3A). Oral administration of the *Schisandra* extract effectively reduced the degree of cartilage destruction when compared with that observed in the PBS control group (Figure 3B). In addition, the OARSI grade and subchondral bone plate thickness in the group treated with the *Schisandra* extract were significantly lower than those in the PBS control group (Figure 3C,D). Moreover, we investigated the expression of COX‐2 using immunohistochemistry. Oral administration of the *Schisandra* extract reduced the expression of COX‐2 in the cartilage of the mouse model of OA (Figure 3E,F). Therefore, the above results showed that the *Schisandra* extract inhibited cartilage destruction in a mouse model of OA.

### 3.4 *Schisandra* extract regulated the NF‐κB and MAPK signalling pathway in an in vitro arthritis mimic condition

**FIGURE 4:**
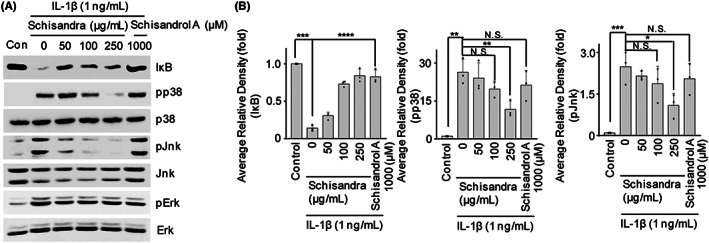
*Schisandra* regulated IL‐1β‐induced the NF‐κB and MAPK signalling pathways. Articular chondrocytes were pretreated with various concentrations of *Schisandra* for 24 h (*n* = 3) before being treated with IL‐1β (1 ng/mL) for 12 min. Protein levels of phosphorylated JNK (pJNK), ERK (pERK) and p38 (pp38) were measured using western blotting. IκB level was detected using western blotting (A) and densitometry (B). Schisandrol A (1000 μM) was used as the positive control. Data were analysed using one‐way analysis of variance with Bonferroni's test, and the plotted values are indicated as mean ± standard error of the mean; *p < 0.05, **p < 0.01, ***p < 0.001 and ****p < 0.0001 compared to the control group.

Articular chondrocytes were pretreated for 24 h in the presence or absence of the *Schisandra* extract and treated with IL‐1β for 15 min before harvesting. Activation of the NF‐κB signalling pathway was assessed based on the degree of IκB degradation. IL‐1β‐induced IκB degradation was blocked by the *Schisandra* extract, as determined using western blotting (Figure 4A) and densitometric analysis (Figure 4B). In articular chondrocytes, IL‐1β‐induced phosphorylation of JNK and p38 was inhibited by treatment with *Schisandra* extract. However, the induced phosphorylation of ERK was not affected by treatment with the safflower seed extract in articular chondrocytes. Taken together, these results indicated that the *Schisandra* extract inhibited the pathogenesis of OA by suppressing the NF‐κB and MAPK signalling pathways by blocking IκB degradation and p38 and JNK phosphorylation.

### 3.5 *Schisandra* extract regulated the NF‐κB and MAPK signalling pathway‐related genes more than schisandrol A in RNA‐sequencing (seq) analysis in an in vitro arthritis mimic condition

**FIGURE 5:**
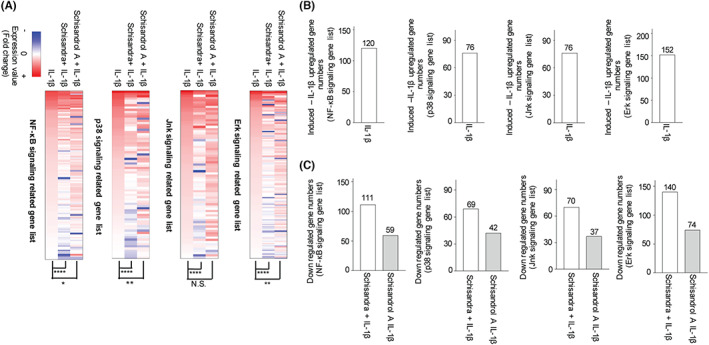
*Schisandra* regulated IL‐1β‐induced NF‐κB and MAPK signalling pathway‐related genes more than schisandrol A in RNA‐sequencing (seq) analysis. RNA‐seq analysis data was used to construct the heat map and it was visualized using Prism. (A) The related gene numbers were quantified and plotted using heat map data. (B, C) Data were analysed using one‐way analysis of variance with Bonferroni's test, and the plotted values are indicated as mean ± standard error of the mean; *p < 0.05, **p < 0.01, ***p < 0.001, and ****p < 0.0001 compared to the control group.

**FIGURE 6:**
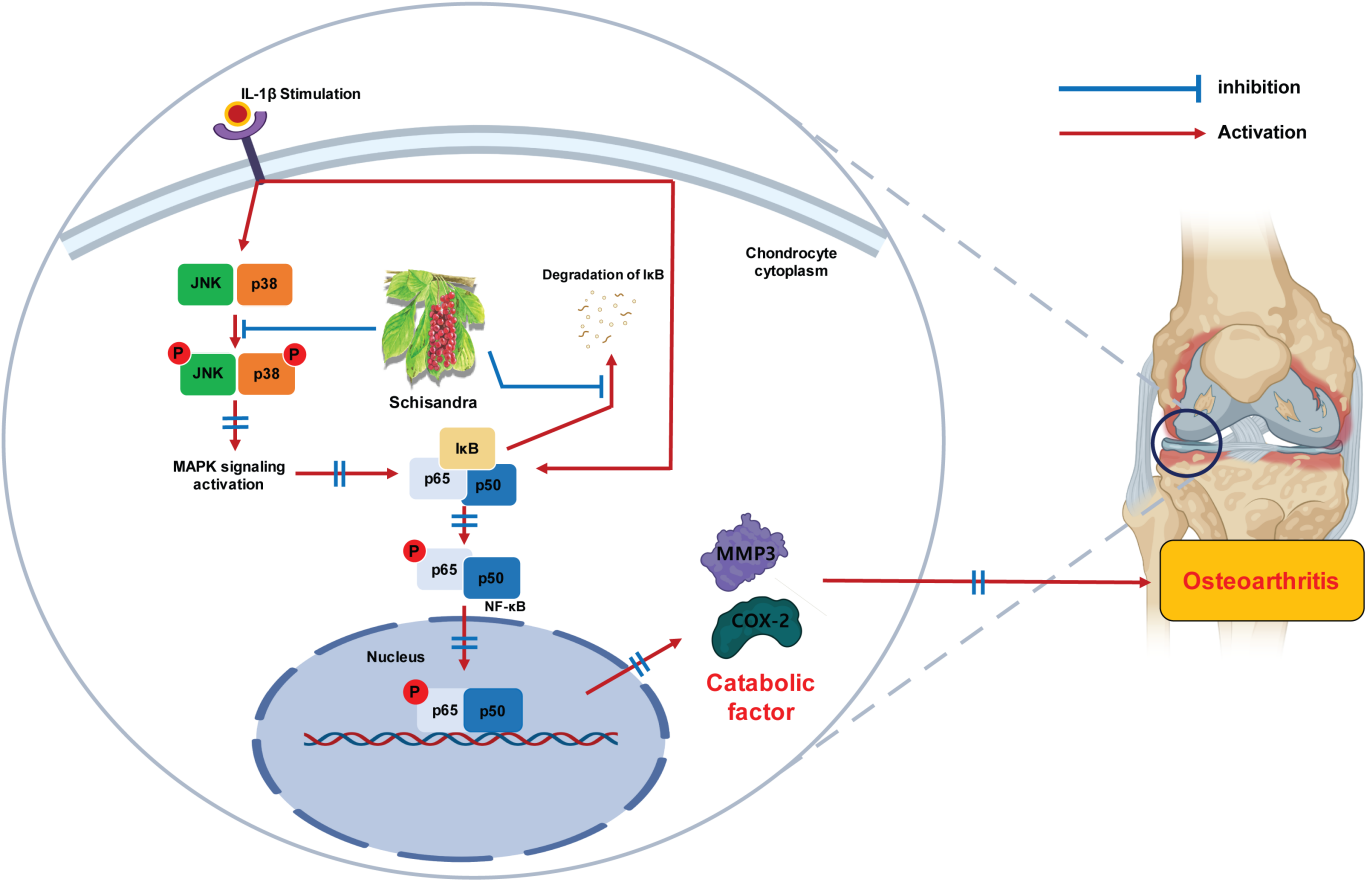
A general summary of the findings: the *Schisandra chinensis* extract improved arthritis pathogenesis by inhibiting the IL‐1β‐induced NF‐κB and MAPK signalling pathways, thereby significantly reducing the expression levels of MMP3 and COX‐2.

Figure 4 shows that *Schisandra* regulates the IL‐1β‐induced increase in the expression of the MAPK and NF‐κB signalling pathway genes. We analysed how the expression pattern of these genes was altered by the *Schisandra* extract and schisandrol A. Lists of genes involved in each signalling pathway were derived from Ingenuity Pathway Analysis (IPA), and the RNA‐seq data prepared using each gene list after treatment of IL‐1β‐stimulated chondrocytes with the *Schisandra* extract and schisandrol A were compared to obtain the pattern of gene expression (Figure 5A). We observed that the expression of the NF‐κB and MAPK signalling pathway (p38, JNK and ERK)‐related genes, which was increased by IL‐1β treatment, decreased considerably when the *Schisandra* extract was used compared to when schisandrol A alone was used (Figure 5B,C). Thus, we suggest that the *Schisandra* extract may potentially inhibit IL‐1β‐activated MAPK and NF‐κB signalling pathways more than schisandrol A (Figure 6).

## 4 DISCUSSION

As damaged cartilage in OA cannot regenerate completely, painkillers with multiple negative effects are the mainstay of therapy.^26^, ^27^, ^28^ Therefore, there is an urgent need for safe and efficient drugs for OA treatment. Recent reports have suggested the use of extracts from natural herbs or their components for treating OA.^29^, ^30^ Therefore, this study was conducted to confirm the possibility of using *Schisandra* extract as a potential treatment for OA.

Several studies have found that herbal extracts alleviate chondrocyte inflammation, control pain and improve joint function.^30^, ^31^ Indeed, plant‐derived components with few side effects and significant pharmacological effects have attracted attention as ideal drugs for OA. Several countries, including Korea, Russia and China, have used *S. chinensis* fruits to treat night sweats, dry cough, insomnia, asthma, involuntary ejaculation, urinary disorders, poor memory, chronic diarrhoea, hyperacidity, diabetes and hepatitis.^32^ Several researchers have reported that the *S. chinensis* fruits have neuroprotective, anti‐inflammatory and antioxidative effects.^33^, ^34^ The main functional constituents of *S. chinensis* are schisandrol A (schisandrin), schisandrol B (gomisin A), deoxyschisandrin (schisandrin A) and γ‐schisandrin (schisandrin B).^35^ Schisandrol B has been shown to inhibit the inflammatory response of microglia to lipopolysaccharide by inhibiting the NF‐κB/MAPK pathway.^36^ Schisandrol A inhibits OA progression via the NF‐κB signalling pathway.^25^ In addition, several studies have shown that Schisandrin A and B can inhibit cartilage degradation by inhibiting the NF‐κB and MAPK signalling pathway.^37^, ^38^ Other studies have shown that the *Schisandra* leaf extract blocks inflammatory effects and could be useful for treating OA.^22^, ^23^ In the present study, oral administration of the *Schisandra* extract protected against cartilage breakdown in a DMM mouse model of OA.

Recent reports revealed that IL‐1β controls the expression of catabolic factors in mouse chondrocytes.^39^, ^40^ Therefore, experimental OA conditions can be simulated in vitro using mouse chondrocytes. IL‐1β treatment of chondrocytes increases the expression of catabolic factors, including MMP3 and COX‐2.^41^, ^42^ These proteases contribute to collagenase activity and the breakdown of type II collagen after cartilage destruction.^43^ Prostaglandin synthesis mediated by COX‐2 is a principal player of OA, and elevated levels of prostaglandins upregulate MMP synthesis.^44^, ^45^ Clinical evidence demonstrates that MMP and COX‐2 are highly expressed in the cartilage of patients with arthritis, which causes OA.^46^ In this study, the *Schisandra* extract decreased the expression of MMP3, COX‐2 and PGE_2_ induced by IL‐1β stimulation in chondrocytes.

NF‐κB and MAPK signalling play crucial roles in cellular responses to various factors, including stress, chemokines and pro‐inflammatory cytokines,^47^, ^48^ and are critical for the progression of OA. Activation of NF‐κB signalling is initiated by the degradation of the IκB protein inhibitor bound to NF‐κB.^49^ After IκB is degraded in response to numerous stimuli, p65, one of the subunits of the NF‐κB complex, is phosphorylated, following which the NF‐κB complex translocates to the nucleus, wherein it upregulates MMPs and COX‐2, targets of NF‐κB.^50^, ^51^ The downstream players of the MAPK signalling pathways include p38, JNK and ERK1/2. Studies have shown that IL‐1β induces the phosphorylation of ERK, JNK and p38, which activate the transcription factors associated with MAPK signalling.^51^, ^52^ This is involved in the production of inflammatory factors, including COX‐2 and MMPs.^53^ Several studies have shown that the activation of the NF‐κB and MAPK signalling pathways increases the expression of catabolic factors that disrupt joint cartilage, leading to OA progression.^41^, ^53^ In addition, the MAPK signalling pathway stimulates the activation of NF‐κB by inducing the phosphorylation of IκB.^54^ Previous studies have established that *Schisandra* has the ability to suppress the NF‐κB signalling pathway and inhibit the phosphorylation of JNK and p38, while leaving the phosphorylation of ERK unaffected. This leads to a suppression of catabolic factors induced by IL‐1β.^55^ Consistent with these findings, our study demonstrated that treatment with *Schisandra* extract in IL‐1β‐stimulated chondrocytes resulted in the inhibition of JNK and p38 phosphorylation, alongside the blocking of the NF‐κB signalling pathway. Additionally, it has been reported that the MAPK signalling pathway induces phosphorylation of IκB, resulting in its degradation and the subsequent activation of the NF‐κB signalling pathway.^54^ Hence, suppressing MAPK signalling can effectively inhibit NF‐κB signalling. Overall, our study revealed that treatment with *Schisandra* extract blocked IκB degradation and repressed phosphorylation of both p38 and JNK, without affecting ERK phosphorylation. Consequently, both the MAPK and NF‐κB signalling pathways were inhibited.

As described previously, IL‐1β activates the MAPK and NF‐κB signalling pathways.^51^, ^52^ Using IPA analysis on RNA‐seq data, the pattern of changes in the expression of NF‐κB and MAPK signalling‐related genes in chondrocytes under in vitro OA conditions, which were increased by IL‐1β, was analysed. We found that the number of genes, whose expressions were elevated by IL‐1β, decreased further when treated with the *Schisandra* extract than with schisandrol A. RNA‐seq is a widely used method for analysing mRNA expression levels. While mRNA expression levels are assumed to correspond proportionally with protein expression levels, previous studies highlighted instances where this relationship may not hold true. Factors such as post‐transcriptional modification, complex gene regulation mechanisms and various biological factors can contribute to such discrepancies.^56^, ^57^, ^58^, ^59^ As a result, we aimed to confirm changes in ERK‐related pathway genes at the RNA expression level. However, it is important to note that the expression of certain genes and their corresponding proteins may differ. This explains the lack of change observed in PERK protein expression levels. However, our results collectively indicate that *Schisandra* extract has the potential to block the activation of both the MAPK and NF‐κB signalling pathways to a greater extent than schisandrol A alone. In conclusion, our study demonstrated that oral administration of *Schisandra* minimizes the progression of OA by preventing cartilage disruption. Moreover, *Schisandra* decreased the expression of catabolic factors (MMP3 and COX‐2) by blocking MAPK and NF‐κB signalling. Schisandrol A, one of the various active compounds from the *Schisandra* extract, affected MAPK signalling and NF‐κB‐related genes more than schisandrol A alone. Therefore, the inhibitory effect of *Schisandra* extract on OA progression can be higher than that of single compounds. We suggest that the preparation of therapeutic agents using not only the *Schisandra* extract but also natural extracts containing various active compounds may be potentially used to treat OA. Furthermore, some studies have demonstrated the use of *S. chinensis* extract in clinical trials and applications for various diseases.^60^, ^61^ Therefore, further research is needed to confirm its efficacy in treating patients with OA. Hence, we anticipate that through the outcomes of this study and subsequent research endeavours, *S. chinensis* extract may effectively mitigate the underlying pathogenesis of human OA. In brief, our study suggests that the utilization of *Schisandra* extract holds promise as a potential drug worthy of investigation, capable of providing an effective treatment for various diseases associated with the NF‐κB and MAPK signalling pathways, as well as OA treatment.

## AUTHOR CONTRIBUTIONS

**Seong Jae Han:** Conceptualization (lead); data curation (lead); formal analysis (lead); investigation (lead); validation (lead); writing – original draft (lead). **Hyemi Lee:** Conceptualization (lead); formal analysis (lead); investigation (lead); methodology (lead); validation (lead); writing – original draft (lead). **Jiho Nam:** Conceptualization (supporting); formal analysis (supporting); investigation (supporting); methodology (supporting). **Cheol‐Ho Pan:** Resources (lead). **Jimin Jeon:** Conceptualization (equal); funding acquisition (lead); project administration (lead). **Siyoung Yang:** Conceptualization (lead); formal analysis (lead); funding acquisition (lead); project administration (lead); supervision (lead); writing – original draft (lead); writing – review and editing (lead).

## FUNDING INFORMATION

This work was supported by Korea Institute of Marine Science & Technology Promotion(KIMST) funded by the Ministry of Oceans and Fisheries(20210647), the National Research Foundation funded by the Ministry of Science & ICT (NRF‐2021M3E5E7023855, NRF‐2022R1A2C2004343, NRF‐2022R1A2C1004688, NRF‐2016M3A9D3915857 and RS‐2023‐00223552), KRIBB (KGM1711134081), the National Research Council of Science & Technology (NST) of the Korean government (MSIT) (CRC21021), and the Research Fund of Ajou University School of Medicine.

## CONFLICT OF INTEREST STATEMENT

The authors confirm that there are no conflicts of interest.

## Supporting information


Table S1.

## Data Availability

The data used to support the findings of this study are included within the article.
