# Supplementary material for: Schisandra extract ameliorates arthritis pathogenesis by suppressing the NF‐κB and MAPK signalling pathways
Source: J Cell Mol Med. 2023 Jun 20;27(14):2071–81. doi: 10.1111/jcmm.17814 (PMC10339094; doi:10.1111/jcmm.17814)
Supplement: Supplementary file 1 — Table S1. [file JCMM-27-2071-s001.pdf]

Supplemental Table S1. Primer sequences and PCR conditions.

Gene	Origin	Strand	Sequence	Size (bp)	AT[a] (°C)
Mmp3	Mouse	[b]S	5′-CTGTGTGTGGTTGTGTGCTCATCCTAC-3′	350	58
		[c]As	5′-GGCAAATCCGGTGTATAATTCACAATC-3′		
Cox-2	Mouse	S	5′-GGTCTGGTGCCTGGTCTGATGAT-3′	724	65
		As	5′-GTCCTTTCAAGGAGAATGGTGC-3′		
Gapdh	Mouse	S	5′-TCACTGCCACCCAGAAGAC-3′	450	58
		As	5′-TGTAGGCCATGAGGTCCAC-3′		

[a]AT, annealing temperature; [b]S, sense; [c]As, antisense
